# Distinct Mg²⁺ responses and species-specific transcriptional regulation of the magnesium transporter MGR2 in *Saccharum officinarum* and *Saccharum spontaneum*

**DOI:** 10.1186/s12870-026-09288-x

**Published:** 2026-06-18

**Authors:** Huihong Shi, Dadong Lin, Xiuting Hua, Zhongmou Yu, Zhen Li, Cheng He, Yuhong Lan, Hongyan Ding, Wei Gu, Hai Zhang, Qiutao Xu, Jisen Zhang

**Affiliations:** 1 Guangxi Sugarcane Bio-breeding Laboratory, State Key Laboratory for Conservation and Utilization of Subtropical Agro-Bioresources, College of Agriculture, Guangxi University, Nanning, Guangxi 530004 China; 2 Center for Genomics and Biotechnology, National Sugarcane Engineering Technology Research Center, Fujian Provincial Key Laboratory of Haixia Applied Plant Systems Biology, Fujian Agriculture and Forestry University, Fuzhou, 350002 China

**Keywords:** Gene expression, Gene regulation, Mg²⁺ release transporters, *Saccharum*

## Abstract

**Background:**

Magnesium (Mg²⁺) is essential for chlorophyll synthesis, enzyme activation, and photosynthesis, but the differential regulation of Mg²⁺ homeostasis between the two founding *Saccharum* species (*Saccharum officinarum* and *Saccharum spontaneum*) remains unknown.

**Results:**

We systematically characterized the MGR gene family in *S. spontaneum* and *S. officinarum* and uncovered distinct physiological responses to Mg²⁺ availability between the two species. Comparative expression analysis across the leaf developmental gradient and diurnal cycles revealed species-specific transcriptional dynamics of *MGR2*, suggesting divergent regulatory mechanisms underlying Mg²⁺ management. Functional complementation in the Mg²⁺-deficient *Salmonella typhimurium* MM281 mutant demonstrated that both SsMGR2 and SoMGR2 restore Mg²⁺ uptake, confirming their conserved transport capability. Overexpression of *SsMGR2* in rice conferred increased biomass under Mg²⁺ deficiency and enhanced tolerance to Mg²⁺ excess, indicating a broad role for this gene in Mg²⁺ homeostasis. Promoter architecture and transcription factor prediction further revealed interspecific divergence, with BBX25, COL5, and WRKY19-2 exhibiting species-dependent regulatory interactions that potentially explain the observed differences in *MGR2* expression.

**Conclusion:**

Overall, the research results indicate that *S. spontaneum and S. officinarum* exhibit different Mg²⁺ responses and *MGR2* regulatory patterns, highlighting the distinct strategies for Mg²⁺ homeostasis in these two founding species. This work provides new insights into the transport mechanism of Mg²⁺ in sugarcane and preliminarily identifies candidate genes and regulatory factors for improving nutrient efficiency and stress recovery ability.

**Supplementary Information:**

The online version contains supplementary material available at https://doi.org/10.1186/s12870-026-09288-x.

## Introduction

Magnesium (Mg²⁺) is the eighth most abundant element in the Earth’s crust and the second most prevalent cation in plant cells after potassium [1]. As the central atom of chlorophyll, Mg²⁺ is indispensable for light energy capture and the initiation of photosynthetic electron transport [2, 3]. It also acts as a key enzyme cofactor for carbon fixation, helping to stabilize Rubisco’s active structure and improve its ability to recognize CO₂ [4–6]. Beyond photosynthesis, Mg²⁺ forms complexes with ATP to sustain cellular energy metabolism and acts as a structural bridge in ribosome assembly, thereby reinforcing ribosomal integrity and protein synthesis [7–10]. By modulating phloem sucrose loading, Mg²⁺ further regulates carbohydrate partitioning from source to sink tissues [11–13]. Adequate Mg²⁺ levels are therefore critical for normal plant growth; deficiency leads to reduced yield.

Plants must tightly regulate Mg²⁺ homeostasis. In plant cells, free Mg²⁺ is maintained at 0.2–1.0 mmol/L [14]. After uptake by roots, Mg²⁺ is loaded into the xylem and transported to shoots via the transpiration stream, followed by redistribution among tissues [15]. These long- and short-distance Mg²⁺ fluxes depend on specific transport proteins and channels [16]. Mg²⁺ first passes through the plasma membrane via a specific transporter, and then is distributed to the organelles. The transporters on the organelle membranes maintain the intracellular Mg²⁺ homeostasis within the organelles [17]. Two main types of Mg²⁺ transport systems have been identified, including the Mg²⁺/H⁺ antiporter MHX1 [18] and the CorA-like MGT/MRS2 family, which is well characterized in higher plants [1, 19]. In particular, the MGT protein family, which includes members such as NIPA, CorA, and MRS2, plays an essential role in maintaining magnesium ion homeostasis in plants [4, 20]. These MGT genes are predominantly expressed in root tissues and are involved in magnesium uptake, translocation, maintenance of ionic balance within the cellular sap, and the accumulation of magnesium ions in the cytoplasm [21–23]. The MGT family has been preliminarily characterized in both *S. spontaneum* and *S. officinarum* [24]. Furthermore, there exists a Mg²⁺ release transporter protein (MGR) family in plants that is homologous to “ancient conserved domain proteins” (ACDPs). This family is named after its role in mediating the release of magnesium ions in the root steles [25, 26]. In *Arabidopsis*, nine MGR proteins (AtMGR1–AtMGR9) have been shown to transport Mg²⁺ and maintain cellular Mg²⁺ homeostasis but differ in subcellular localization and mechanism [27]. AtMGR1–AtMGR3 localize to the tonoplast and promote vacuolar Mg²⁺ storage [28]. AtMGR4–AtMGR7 are plasma membrane localized and facilitate Mg²⁺ translocation from roots to shoots [25]. AtMGR8 and AtMGR9 reside in the chloroplast inner membrane and mediate Mg²⁺ uptake into chloroplasts [16]. While MGR functions are increasingly well defined in *Arabidopsis*, their roles in crops remain largely unknown. In sugarcane, understanding how MGR transports Mg²⁺ is essential for improving Mg²⁺ use efficiency and enhancing tolerance to the Mg-deficient soils typical of tropical regions.

Sugarcane is a pivotal sugar and energy crop in tropical and subtropical regions [29], where intense rainfall often causes substantial Mg²⁺ leaching and low soil Mg availability [30, 31]. Under such conditions, Mg²⁺ deficiency severely restricts sugarcane growth and development [32]. Integrated and balanced fertilization with nitrogen, phosphorus, potassium, sulfur, and magnesium significantly improves sugarcane yield and quality [33], underscoring the central role of Mg in determining stalk number, internode elongation, and sucrose content [11, 30, 32]. Modern cultivars are derived from interspecific hybrids between *Saccharum spontaneum* and *Saccharum officinarum*, followed by repeated backcrosses to *S. officinarum* [34, 35]. *S. spontaneum* and *S. officinarum* differ markedly in photosynthetic efficiency, sucrose accumulation, and stress tolerance. The functions and regulatory mechanisms of the MGR gene family in sugarcane remain unexplored. In this study, we systematically identified *MGR* genes in *S. spontaneum* and *S. officinarum*, with particular emphasis on functional characterisation of *SsMGR2* and *SoMGR2*. We verified their Mg²⁺ transport activity and identified candidate TFs regulating their expression, thereby providing a first insight into the regulatory basis of differential *MGR2* expression between the two species and a theoretical foundation for improving Mg^2+^ use efficiency, yield stability, and sucrose productivity in sugarcane.

## Materials and methods

### Experimental materials

*Saccharum spontaneum* (SES‑208, 2*n* = 8*x* = 64) and *Saccharum officinarum* (LA‑Purple, 2*n* = 8*x* = 80) were cultivated in the sugarcane germplasm repository of Fujian Agriculture and Forestry University under identical agronomic conditions: A photosynthetic photon flux density of approximately 350 µmol m⁻² s⁻¹, a photoperiod of 14 h light/10 h dark, day/night temperatures of 30 °C/22 °C, and relative humidity around 60% [36].

### Identification and physicochemical property analysis of members of the MGR gene family

To identify members of the MGR gene family, protein sequences from a diverse set of species -including *S. bicolor*, *Z. mays*, *S. viridis*, *B. distachyon*, *O. sativa*, *A. comosus*, *C. papaya*, *A. thaliana*, *P. trichocarpa*, *V. vinifera*, *S. lycopersicum*, *A. trichopoda*, *S. moellendorffii*, *C. reinhardtii*, and *D. salina* -were obtained from the Phytozome portal (https://phytozome-next.jgi.doe.gov/). These *A. thaliana* MGR proteins were then used as queries BLASTp searches against the genomes of *S. spontaneum* and *S. officinarum*, applying an E‑value threshold of ≤ 1 × 10⁻⁵ to retrieve candidate homologs. The identified sequences were screened for the presence of DUF21 (PF01595) and CBS (PF00571) domains using HMMER (hmmsearch) [37]. All hits were subsequently validated with the SMART (http://smart.embl.de/smart/set_mode.cgi?NORMAL=1#) [38] and NCBI‑CDD (https://www.ncbi.nlm.nih.gov/Structure/cdd/wrpsb.cgi ) databases to confirm domain architecture and to delineate the *S. spontaneum* and *S. officinarum MGR* gene groups. Homologous sorghum genes were used as references to assign systematic nomenclature to the sugarcane *MGR* genes. Finally, subcellular localization was predicted using the Softberry platform (http://linux.softberry.com/). The relative molecular mass, isoelectric point and amino acid number of the MGR proteins in sugarcane were analyzed using ExPasy (https://www.expasy.org/) [39].

### Evolutionary analysis of *MGR* genes

Nuclear genome‑encoded MGR protein sequences from seventeen representative species were aligned using the ClustalW algorithm in MEGA7 [40]. The resulting alignment was used for phylogenetic reconstruction using two methods. A neighbor‑joining (NJ) tree was constructed under a Poisson substitution model with pairwise deletion of gaps, and node support was assessed with 1 000 bootstrap replicates. Concurrently, a maximum‑likelihood (ML) tree was independently generated with IQ‑TREE, employing automatic thread allocation (‑nt AUTO) and ultrafast bootstrap approximation (‑bb 1000) to evaluate node confidence. Both phylogenetic trees were subsequently visualized using the interactive Tree Of Life (iTOL) web platform (https://itol.embl.de/ ) [41].

### *K*_a_/*K*_s_ analysis and estimated divergence time for *MGRs*

The ratios of non-synonymous substitution (*K*_a_) to synonymous substitution (*K*_s_) rates were calculated by the easy_Kaks calculation program (https://github.com/tangerzhang/FAFU-cgb/blob/master/easy_KaKs), employing Fisher’s exact test for the statistical validation of the *K*_a_ and *K*_s_ estimates. The divergence time (T) for each gene pair was then estimated using the formula T = *K*_s_ / (2 × 6.1 × 10^− 9^) × 10^− 6^ million years ago (Mya) [42].

### Gene expression pattern analysis and correlation analysis

Gene expression patterns of MGR family members and associated TFs were analyzed using transcriptome data from *S. spontaneum* and *S. officinarum*, encompassing gradient-developing leaves, various stem and leaf tissues at distinct developmental stages, and mature leaves under diurnal cycles (data available at https://sugarcane.gxu.edu.cn/scdb/). The correlation coefficient (*r*) between the expression of transcription factors and *SsMGR2* or *SoMGR2* was calculated using the CORREL function at the gradient-developing leaves and diurnal cycles, respectively. Student’s t-test was used for significance analysis to calculate the *P*-value. “*P* < 0.05 was considered statistically significant.” Visualization was performed using the ggplot2 package (https://github.com/tidyverse/ggplot2).

### RT-qPCR verification

We took the first leaf of the sugarcane, with three leaves as one biological repeat, and set up three biological repeats and three technical repeats. Total RNA was extracted from sugarcane leaves using the FreeZol Reagent (Vazyme, item R711-01) according to the manufacturer’s protocol. Subsequently, first-strand cDNA was synthesized from the purified RNA using the StarScript II First-strand cDNA Synthesis Kit with gDNA Remover (Genstar, A222-10). Quantitative real-time PCR (RT-qPCR) was then performed following the kit’s instructions. Gene-specific primers were designed with the NCBI Primer-BLAST online tool (https://www.ncbi.nlm.nih.gov/tools/primerblast/index.cgi?LINK_LOC=BlastHome). The product was purchased from GenStar (A310-02). The *WDR* (WD repeat-containing protein) (*Sspon.06G0012080*) and *OsUBQ5* (*Ubiquitin 5*) were used as housekeeping genes. The reaction mixture contained 10 µL of 2 × Real Star Green Fast Mixture, 0.5 µL of 50 ng/µL cDNA template, 0.5 µL of 10 mM forward primer, 0.5 µL of 10 mM reverse primer, and 8.5 µL of RNase-free H₂O. Real-time PCR was performed using a BioRad CFX Connect Real-Time system under the following conditions: initial denaturation at 95 °C for 2 min, followed by 40 cycles of 95 °C for 15 s, 60 °C for 15 s, and 72 °C for 30 s. The relative expression level of the SsMGR2 and SoMGR2 were calculated using the 2^⁻ΔΔCt^ method [43]. Details of the primers were shown in Supplementary Table 1.

### MgSO_4_ treatment experiment

The sugarcane materials used in this experiment consisted of seedlings grown from stem nodes. The nodes were washed, soaked for 24 h, then transplanted into nutrient soil for 20 days. To deplete the endogenous magnesium ion content, the seedlings were subsequently treated with tap water for 5 days, followed by 5 days of treatment with distilled water. Uniform seedlings were selected and transferred to hydroponic solutions containing different MgSO₄ concentrations (0 mM, 0.02 mM, 2 mM, 5 mM, and 10 mM). Each treatment included six biological replicates per variety, and the nutrient solution was refreshed every 7 days. Phenotypic observations were recorded after 40 days of treatment. In parallel, 30-day-old rice seedlings were subjected to the same MgSO₄ treatment regimen and observed after 45 days. Chlorophyll content was measured using a SPAD-502 Plus chlorophyll meter (Konica Minolta, Japan). The fresh weight of the seedlings was recorded under different Mg²⁺ concentrations, with three biological replicates for each treatment group.

### Functional complementary analysis of defective bacteria in the *MGR2* of sugarcane

The coding sequences (CDS) of *SsMGR2* and *SoMGR2* were amplified from cDNA of *S. spontaneum* and *S. officinarum*, respectively, and cloned into the pTrc99A expression vector. The recombinant plasmids were introduced into *Salmonella typhimurium* MM281, a magnesium transport-deficient strain. The transformation process began by streaking the bacteria onto LB agar plates supplemented with 50 µg mL⁻¹ ampicillin, 34 µg mL⁻¹ chloramphenicol, and 20 mM MgCl₂, followed by incubation inverted at 37 °C for 12 h. Single colonies from the above plates were then inoculated into 2 mL of LB broth containing the same antibiotics and grown to late‑log phase. The cultures were subsequently diluted 1:50 into 100 mL of LB medium with the same antibiotic concentrations and cultured at 37 °C with shaking at 200 rpm until the optical density at 600 nm (OD₆₀₀) reached 0.3–0.5. Cells were chilled on ice for 10 min, harvested by centrifugation (4 °C, 4000 × g for 15 min), and the supernatant discarded. The pellet was gently resuspended in 10 mL of pre‑chilled 0.1 M CaCl₂–MgCl₂ solution and incubated on ice for 15 min, before undergoing another centrifugation at 4 °C for 10 min at 4000 × g. After removing the supernatant, 3 mL of pre‑chilled 15% glycerol containing 0.1 M CaCl₂–MgCl₂ was added, and the suspension was mixed gently by pipetting. The mixture was kept on ice for 5 min to generate electrocompetent MM281 cells. The recombinant pTrc99A-SsMGR2 and pTrc99A-SoMGR2 plasmids were introduced into the competent MM281 cells by heat‑shock transformation. Transformants were plated on LB agar supplemented with 10 mM Mg²⁺, 34 µg mL⁻¹ chloramphenicol, and 50 µg mL⁻¹ kanamycin, and incubated overnight at 37 °C. PCR‑confirmed colonies were inoculated into LB broth with the same antibiotics and 10 mM Mg²⁺, grown to OD₆₀₀ = 0.6, and induced with 50 µM isopropyl β‑D‑1‑thiogalactopyranoside (IPTG) for target gene expression. After induction, cultures were adjusted to an OD₆₀₀ of 1.0, serially diluted 10‑fold, and 2 µL of each dilution was spotted onto N‑minimal agar plates containing 0.01, 0.1, 0.5, 1, 5, and 10 mM MgSO₄. Plates were incubated at 37 °C for 48 h [22], and colony growth was documented photographically.

### Heterologous overexpression in rice

The CDS of *SsMGR2* was cloned into the pCXUN-FLAG vector, and the recombinant vector was transformed into rice (*Oryza sativa L*. ssp. japonica) cultivar Zhonghua 11 (ZH11) via Agrobacterium-mediated transformation, which was performed by Wuhan Boyuan Biotechnology Company. For molecular characterization, genomic DNA was extracted from T1 generation plants using a DNA extraction kit. Transgenic lines were identified by PCR amplification using gene-specific primers. Total RNA was isolated from positive transgenic plants using an RNA extraction kit, and the expression level of *SsMGR2* was quantified by RT-qPCR to confirm successful overexpression.

### Promoter sequence extraction and cis-acting element analysis

The 2 kb genomic sequences upstream of the transcription start sites of *SsMGR2* and *SoMGR2* were extracted from the respective *S. spontaneum* and *S. officinarum* genome assemblies using TBtools, based on the annotation information provided in the GFF3 files. Putative cis-acting regulatory elements within these promoter regions were subsequently identified using the online database PlantCARE (http://bioinformatics.psb.ugent.be/webtools/plantcare/htmL/).

### Yeast single-hybrid sieve library and one-to-one validation

The promoters (2 kb) of *SsMGR2* and *SoMGR2* were each truncated into 5 bait fragments, which were respectively cloned into the pAbAi empty vector to construct the pAbAi-bait recombinant vector. The recombinant vector plasmids were linearized by restriction endonuclease Bstb I (R0519V, NEB) and transformed into Y1HGlod yeast competent cells. The transformed yeast was spread on the SD-Ura medium and cultured inverted at 30 °C for 2–3 days. Positive single colonies were picked for the screening of the minimum AbA inhibitory concentration. The yeast libraries were screened for the fragments that could be successfully inhibited by AbA. The specific method is referred to [44]. The positive yeast was extracted with the yeast plasmid extraction kit. The plasmid from the positive yeast clone was extracted using the yeast plasmid mini-extraction kit (D1160, Solarbio) and transformed into *Eschrrichia coli* DH5α competent cells. Then, the plasmid was extracted using the plasmid extraction kit and co-transferred into the Y1HGlod yeast competent cells and spread on the SD-leu-AbA* plate for growth.

### Data analysis

The plotting software used was Origin 2022. The significance analysis was conducted using the Student's *t*-test and Tukey’s HSD test. The symbols * indicate *P* < 0.05, ** indicate *P* < 0.01, and *** indicate *P* < 0.001.

### Result

### Identification and evolutionary analysis of the MGR gene family in *S. spontaneum* and *S. officinarum*

**Fig. 1:**
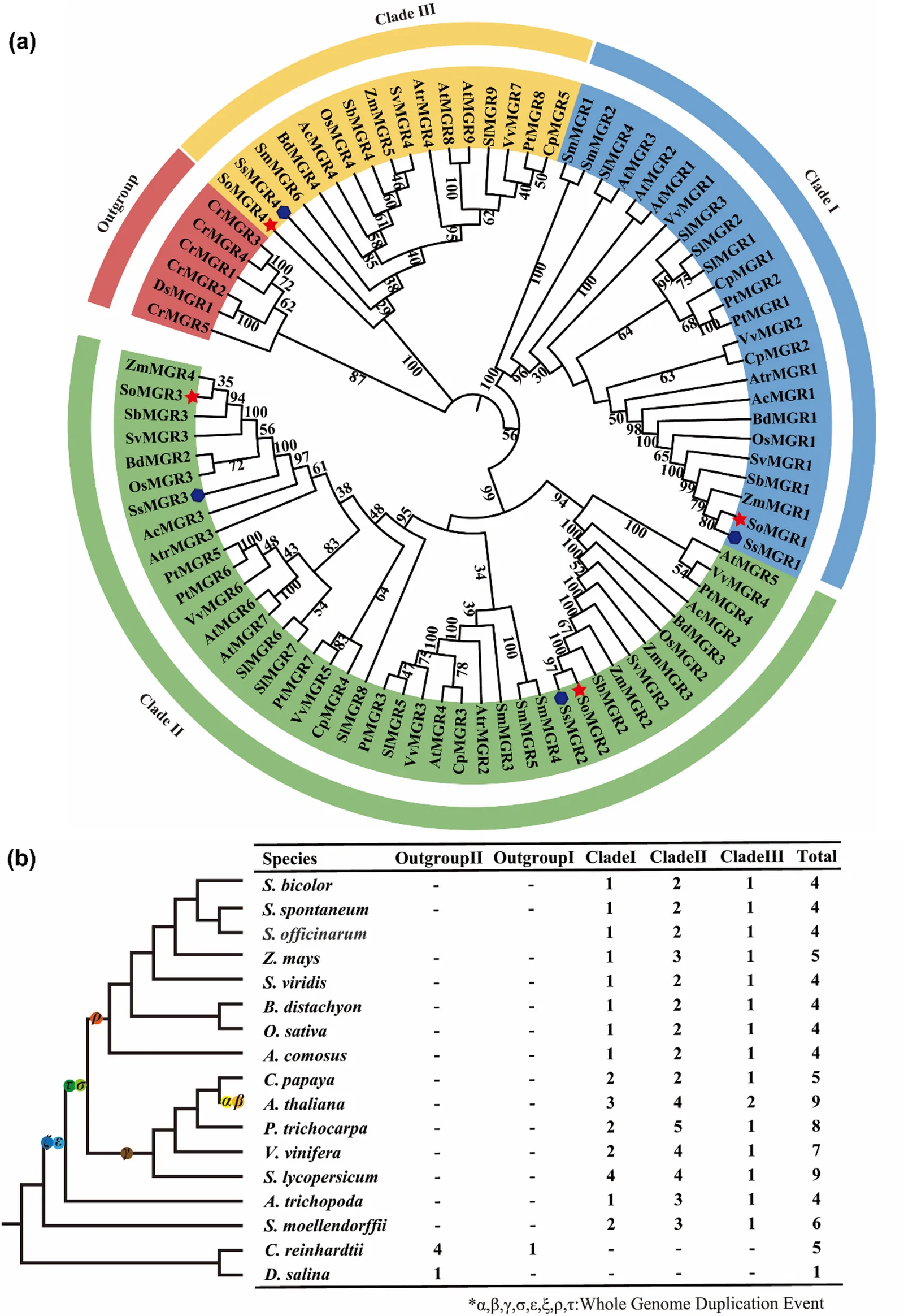
Systematic evolutionary analysis of the *MGR* genes in 17 species. (**a**) The phylogenetic tree of the *MGR* genes of 17 species, including 8 monocotyledonous plants, 7 dicotyledons plants, and 2 eukaryotic unicellular algae organisms. monocotyledonous plants: *Sb* (*S. bicolor*), *Ss* (*S. spontaneum*), *So* (*S. officinarum*), *Zm* (*Z. mays*), *Sv* (*S. viridis*), *Bd* (*B. distachyon*), *Os* (*O. sativa*), *Ac* (*A. comosus*), dicotyledonous plants: *Cp* (*C. papaya*), *At* (*A. thaliana*), *Pt* (*P. trichocarpa*), *Vv* (*V. vinifera*), *Sl* (*S. lycopersicum*), *Atr* (*A. trichopoda*), *Sm* (*S. moellendorffii*), and algae: *Cr* (*C. reinhardtii*), *Ds* (*D. salina*); (**b**) The systematic evolutionary relationship of the *MGR* gene family in 17 species

Based on comparative genomic analyses, four members of the magnesium transporter-related (MGR) gene family were identified in both *Saccharum spontaneum* and *Saccharum officinarum*. These genes were designated *SsMGR1* - *SsMGR4* and *SoMGR1* - *SoMGR4*, respectively, according to their orthology with sorghum *MGR* genes. All identified MGR proteins contained the conserved structural domains characteristic of the MGR family. Physicochemical analysis revealed that MGR proteins from both species ranged from 320 to 530 amino acids in length, with predicted molecular weights of 36.0–58.1 kDa and isoelectric points between 5.6 and 7.72 (Supplementary Table 2). Except for SsMGR3 and SoMGR3, which showed moderate variation in protein length, MGR1, MGR2, and MGR4 exhibited highly similar physicochemical properties between the two species. Subcellular localization prediction indicated that MGR1, MGR2, and MGR3 from both species are predominantly localized to the plasma membrane, whereas MGR4 is predicted to localize to chloroplasts (Supplementary Table 2). These results suggest that the MGR family is structurally conserved between *S. spontaneum* and *S. officinarum*. To elucidate the evolutionary relationships of *MGR* genes, a phylogenetic tree was constructed using 87 MGR protein sequences from 17 plant species, including eight monocots, seven dicots, and two unicellular algae. The MGR proteins clustered into three major clades (Clade I–III; Fig. 1a). Orthologous MGR pairs from *S. spontaneum* and *S. officinarum* consistently grouped within the same clade: MGR1 orthologs in Clade I, MGR2 and MGR3 orthologs in Clade II, and MGR4 orthologs in Clade III. Sugarcane MGR proteins showed the closest phylogenetic relationships with those from other monocot species, particularly sorghum.

Analysis of *MGR* gene copy number across species revealed that the family is broadly conserved, with gene numbers ranging from one to nine (Fig. 1b). In monocot species, *MGR* gene numbers remained relatively stable and were minimally influenced by whole-genome duplication events, whereas dicot species exhibited moderate gene family expansion and greater copy number variation. Furthermore, *K*_a_/*K*_s_ analysis of orthologous *MGR* gene pairs between sorghum and the two sugarcane species yielded values below 1 (0.388–0.958 for *S. spontaneum* and 0.817–0.883 for *S. officinarum*; Supplementary Table 3), indicating that purifying selection has been the predominant evolutionary force shaping the MGR gene family in sugarcane.

### Expression profiling reveals divergent regulation of *MGR2* between *S. spontaneum* and *S. officinarum*

To explore the potential functional roles of *MGR* genes, we analyzed their expression profiles in *S. spontaneum* and *S. officinarum* across multiple tissues, developmental stages, leaf gradient zones, and diurnal cycles (Supplementary Fig. 1). Overall, *MGR1* and *MGR3* displayed largely conserved expression patterns between the two species, with *MGR1* showing relatively high transcript abundance and *MGR3* remaining weakly expressed across tissues and stages. Although *SsMGR4* exhibited higher overall expression levels than *SoMGR4*, their spatial and temporal expression trends were broadly similar.

In contrast, *MGR2* exhibited divergence in expression patterns between the two species. Along the developmental gradient of leaf segments, *SsMGR2* transcripts accumulated predominantly in the transition zone and declined toward the mature zone in *S. spontaneum*, whereas *SoMGR2* followed a different pattern, with higher expression in mature leaf tissues in *S. officinarum* (Supplementary Fig. 1c-d). Diurnal expression analysis further revealed contrasting patterns: *SsMGR2* displayed a distinct rhythmic oscillation, while *SoMGR2* expression remained relatively constant throughout the day-night cycle (Supplementary Fig. 1e-f). These distinct spatiotemporal expression profiles suggest that *MGR2* is subject to species-specific transcriptional regulation and may play divergent physiological roles in magnesium homeostasis between *S. spontaneum* and *S. officinarum*.

### *MGR2* encodes a conserved Mg²⁺ transporter with growth-promoting effects under Mg²⁺ stress

**Fig. 2:**
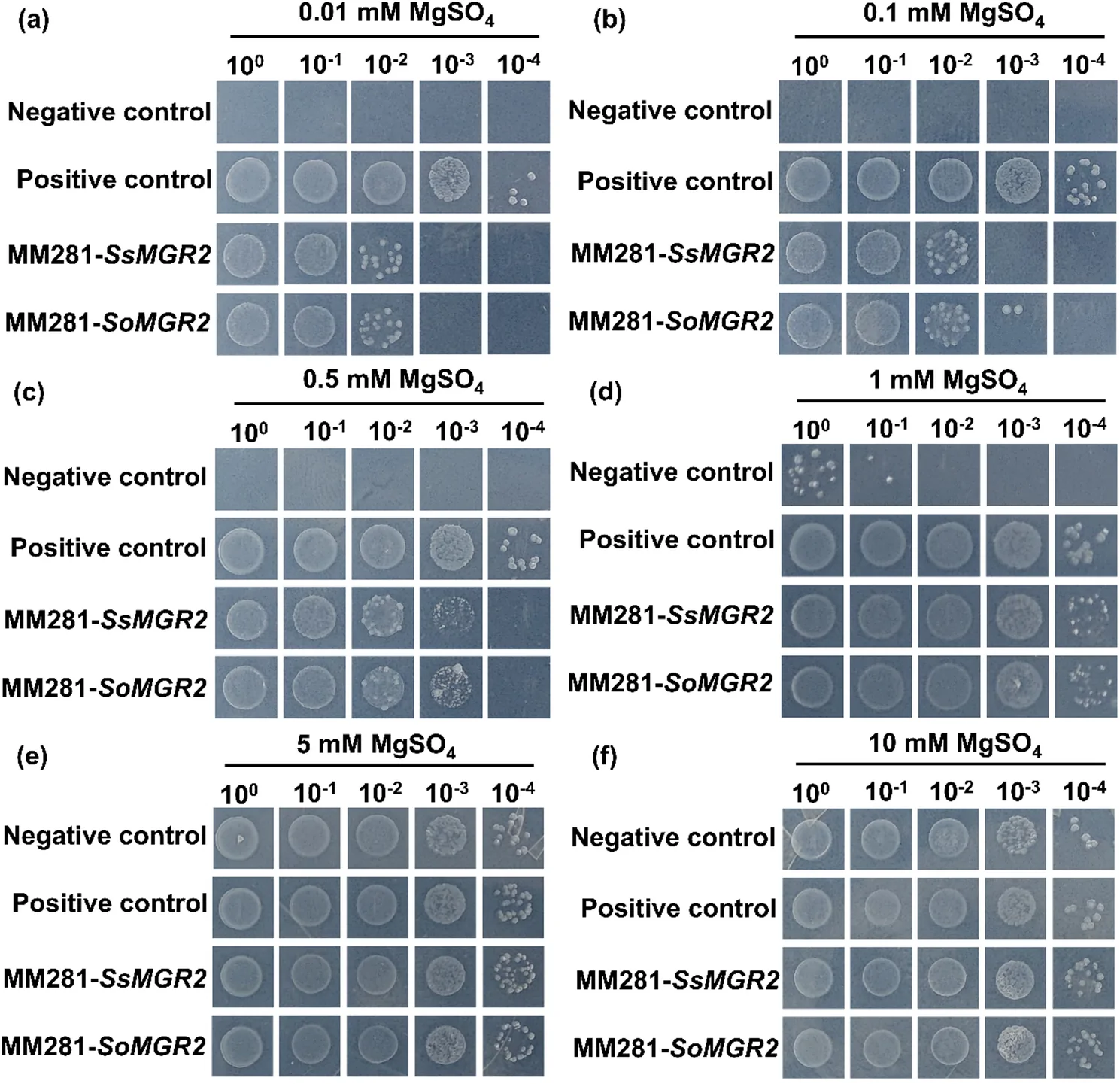
Complementation of the MM281 strains by *MGRs. * Growth phenotypes of MM281 strains in N-minimal medium containing 0.01 mM (**a**), 0.1 mM (**b**), 0.5 mM (**c**),1 mM (**d**), 5 mM (**e**), 10 mM (**f**) MgSO_4_. MM281 strain was transformed with empty vector pTrc99A as a negative control, MM281 strain was transformed with *SsMGT10*-pTrc99A recombinant vector as a positive control. MM281-*SsMGR2* and MM281-*SoMGR2* represent MM281 strains was transformed with *SsMGR2*-pTrc99A and *SoMGR2*-pTrc99A into MM281, respectively

To determine whether MGR2 proteins function as Mg²⁺ transporters, *SsMGR2* and *SoMGR2* were heterologously expressed in the Mg²⁺ uptake–deficient *Salmonella enterica* serovar Typhimurium mutant MM281. The empty vector pTrc99A served as a negative control, while *SsMGT10* was used as a positive control (Fig. 2). Expression of either *SsMGR2* or *SoMGR2* successfully restored bacterial growth over a wide range of MgSO₄ concentrations (0.01-10 mM), demonstrating that both genes encode functional Mg²⁺ transporters. These results indicate that MGR2 transport activity is conserved between the two sugarcane species and that functional divergence at the protein level is unlikely to explain species-specific expression patterns of *MGR2*.

**Fig. 3:**
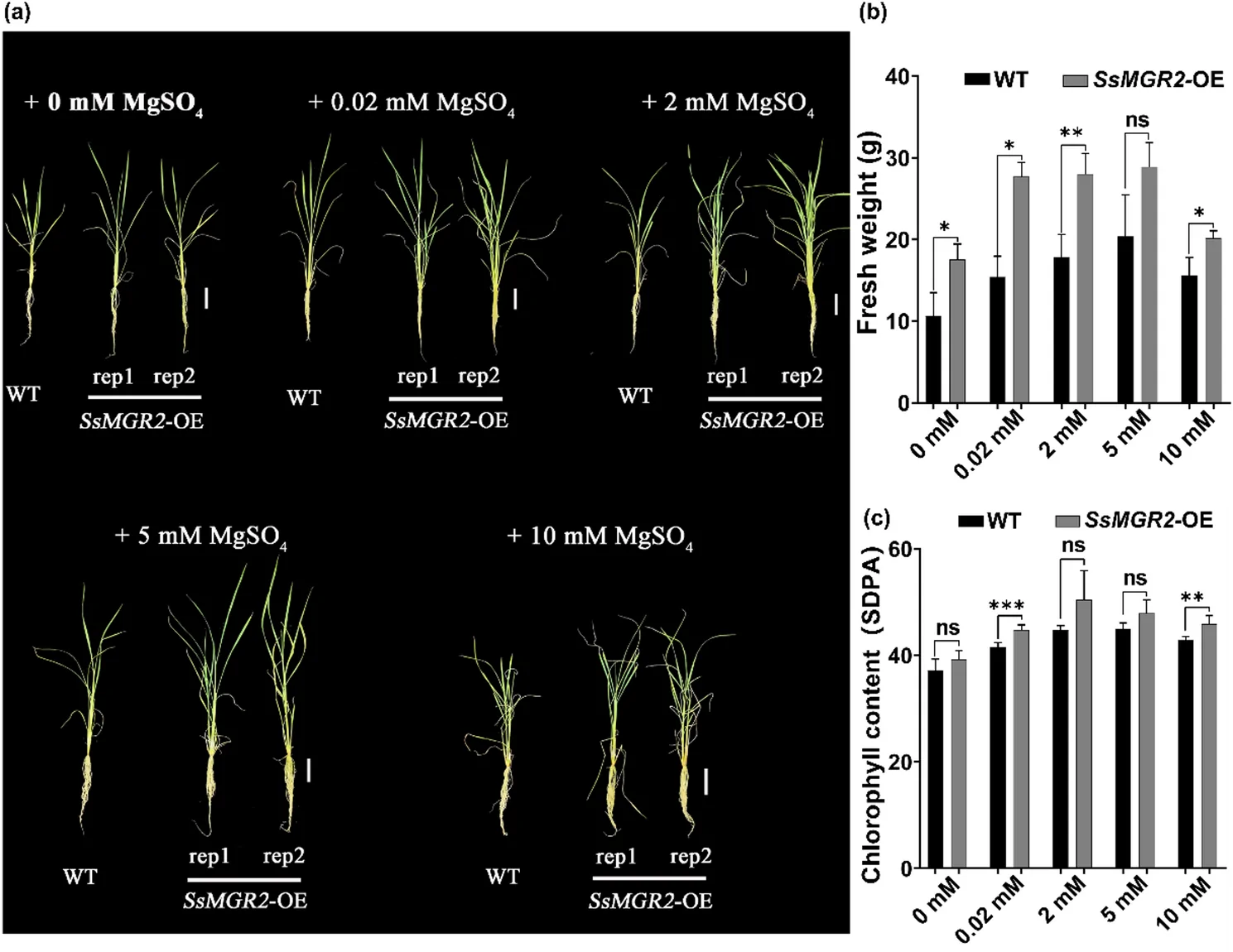
Phenotypes and physiological and biochemical indicators of rice seedlings under different MgSO_4_ treatments. (**a**-**c**) Growth phenotype (**a**), fresh weight (**b**), and chlorophyll content in leaves (**c**) of 45-day-old rice seedlings supplemented with specified Mg^2+^ in magnesium-deficient Hogland nutrient solution. Three biological replicates were set for each treatment. (Student’s *t*-test, “ns” indicates no significant difference, “*” represents “*P* < 0.05”, “**” represents “*P* < 0.01”, and “***” indicates *P* < 0.001)

To further investigate the physiological role of *MGR2* in plants, *SsMGR2* was overexpressed in rice. Under normal growth conditions, *SsMGR2*-overexpressing (OE) lines exhibited increased shoot height and more developed root systems compared with wild-type (WT) plants (Fig. 3a; Supplementary Fig. 2). When exposed to a gradient of MgSO₄ concentrations (0–10 mM), both OE and WT plants exhibited enhanced growth and chlorophyll accumulation at moderate Mg²⁺ levels but were inhibited under excessive Mg²⁺ supply. Notably, OE lines consistently displayed higher fresh weight and chlorophyll content than WT plants across all MgSO₄ treatments. Even under severe Mg²⁺ excess (10 mM MgSO₄), OE plants retained significantly greater biomass and chlorophyll content than WT plants (Fig. 3b–c). These findings demonstrate that *MGR2* positively regulates plant growth and chlorophyll maintenance and enhances tolerance to Mg²⁺ stress.

### Distinct Mg²⁺ responses and *MGR2* transcriptional dynamics in *S. spontaneum* and *S. officinarum*

**Fig. 4:**
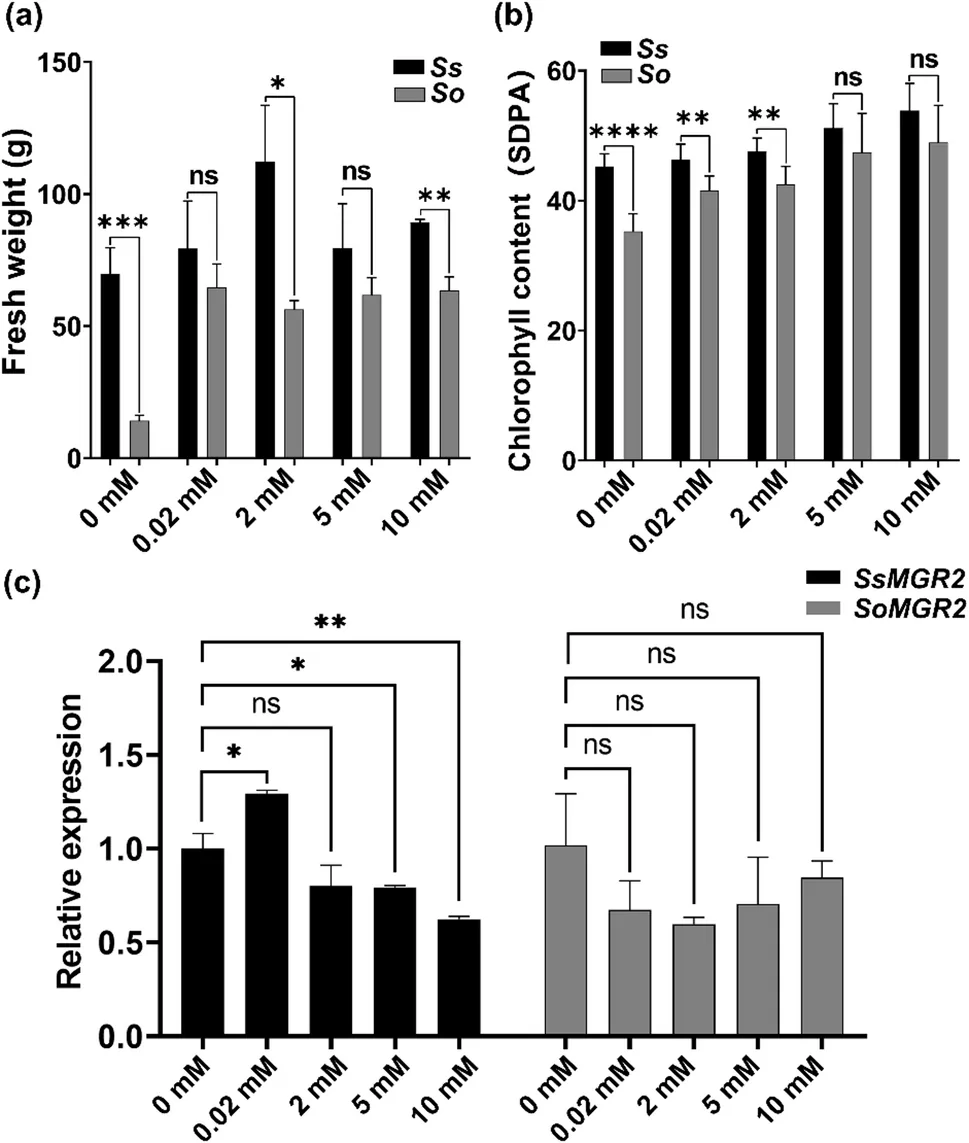
Physiological and biochemical indicators of *S. spontaneum* *(Ss)* and *S. officinarum* (*So)* seedlings under different MgSO_4_ treatments. (**a**) The fresh weight of sugarcane under different concentrations of MgSO_4_ conditions supplemented with magnesium-deficient Hogland nutrient solution; (**b**) The chlorophyll content of sugarcane under different concentrations of MgSO_4_ conditions supplemented with magnesium-deficient Hogland nutrient solution; (**c**) The expression level of the *MGR2* gene after MgSO_4_ treatments

To examine the role of *MGR2* in sugarcane, seedlings of *S. spontaneum* and *S. officinarum* were grown under varying MgSO₄ concentrations (0–10 mM) for 40 days. Under control conditions, *S. spontaneum* exhibited more vigorous root development, greater biomass accumulation, and higher chlorophyll content than *S. officinarum* (Fig. 4a-b). In both species, moderate Mg²⁺ supply promoted growth, whereas excessive Mg²⁺ inhibited biomass accumulation and chlorophyll synthesis.

Notably, *MGR2* transcriptional responses to Mg²⁺ availability differed markedly between the two species. In *S. spontaneum*, *SsMGR2* expression was strongly induced under low Mg²⁺ conditions (0.02 mM MgSO₄) but declined as Mg²⁺ concentrations increased. In contrast, *SoMGR2* expression in *S. officinarum* remained largely unchanged across all MgSO₄ treatments (Fig. 4c). These results suggest that *SsMGR2* is transcriptionally responsive to Mg²⁺ availability and may contribute to efficient Mg²⁺ acquisition and growth promotion in *S. spontaneum*, whereas *SoMGR2* appears to be regulated through a distinct, less Mg²⁺-responsive mechanism.

### Divergent *cis*-regulatory architecture underlies species-specific transcriptional regulation of *MGR2*

**Fig. 5:**
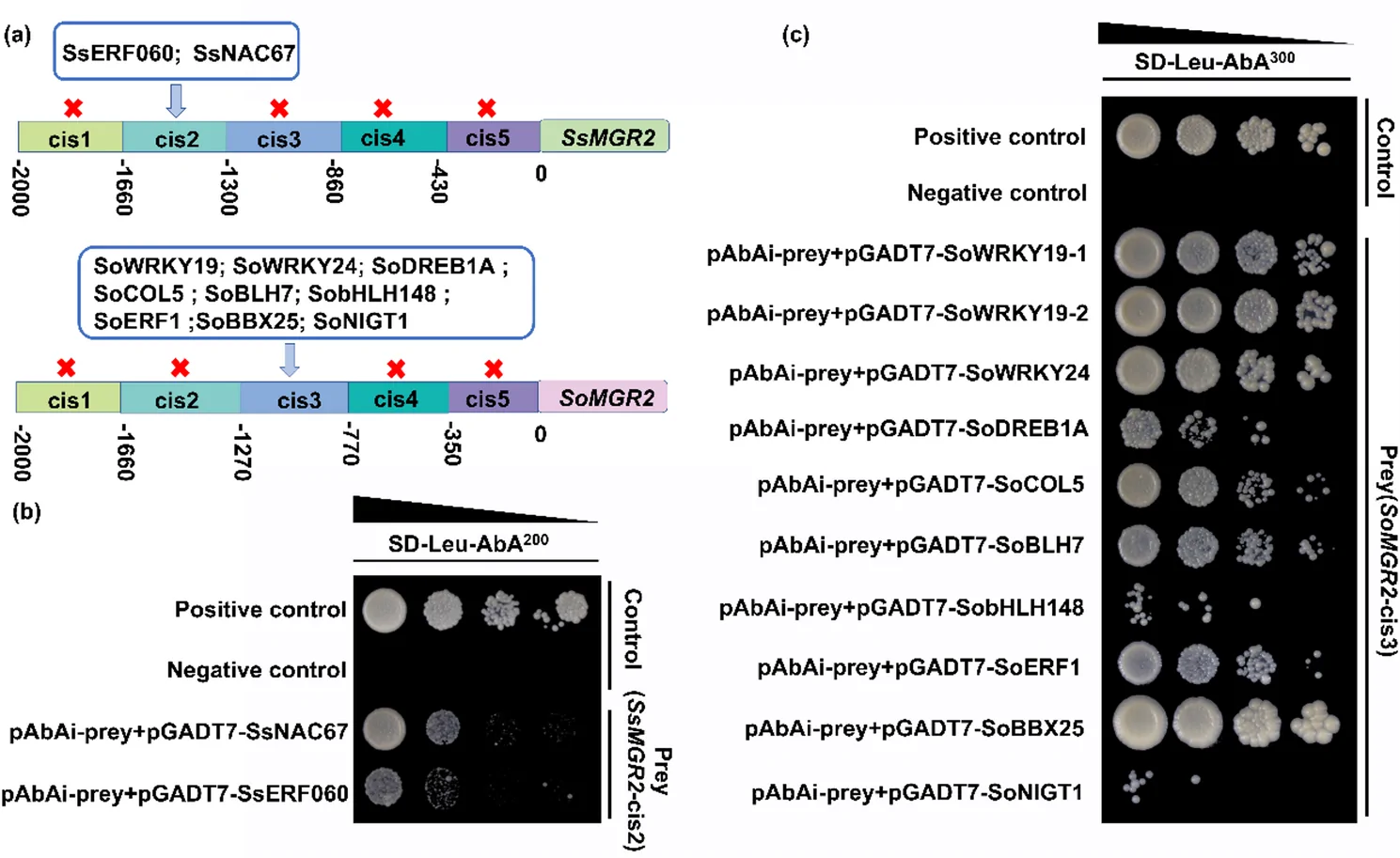
TFs related to *SsMGR2* or *SoMGR2*. (**a**) Schematic diagram of the promoter bait fragments of *SsMGR2* and *SoMGR2*. The 2 kb upstream sequences of *SsMGR2* and *SoMGR2* were respectively divided into cis1-cis5, which were used as bait fragments for screening TFs in the Y1H assays, the blue boxes represent the TFs that were selected from the corresponding bait fragments, while the red “x” indicates the bait fragments that were not successfully inhibited by AbA or did not yield any TFs; (**b**) The one-to-one yeast hybrid results for TFs that potentially interact with the upstream sequences of *SsMGR2*; (**c**) The one-to-one yeast hybrid results for TFs that potentially interact with the upstream sequences of *SoMGR2*

To elucidate the molecular basis of differential *MGR2* regulation, the 2 kb upstream promoter regions of *SsMGR2* and *SoMGR2* were analyzed. A total of 29 cis-acting elements were identified in the *SsMGR2* promoter, compared with 35 elements in the *SoMGR2* promoter (Fig. 5a; Supplementary Fig. 3). In addition to core promoter motifs, both promoters contained multiple hormone-, stress-, and light-responsive elements. However, several cis-elements were uniquely present in each promoter. The *SsMGR2* promoter specifically harbored AE-box, chs-CMA1a, Sp1, CGTCA-motif, TGACG-motif, and GC-motif elements, whereas the *SoMGR2* promoter uniquely contained 3-AF1 binding site, I-box, Pc-CMA2c, TCT-motif, and O2-site elements. These differences indicate substantial divergence in cis-regulatory architecture between the two species.

Promoter truncation assays identified *SsMGR2*-cis2 and *SoMGR2*-cis3 as functional regulatory regions suitable for yeast one-hybrid (Y1H) screening (Fig. 5a; Supplementary Fig. 4). Using these fragments as baits, we identified two transcription factors (TFs) (SsERF060 and SsNAC67) that interact with the SsMGR2 promoter. In contrast, a screen of the *SoMGR2* promoter identified ten TFs, including SoBBX25, SoCOL5, SoDREB1A, and multiple WRKY proteins (Fig. 5b-c; Supplementary Table 4).

**Fig. 6:**
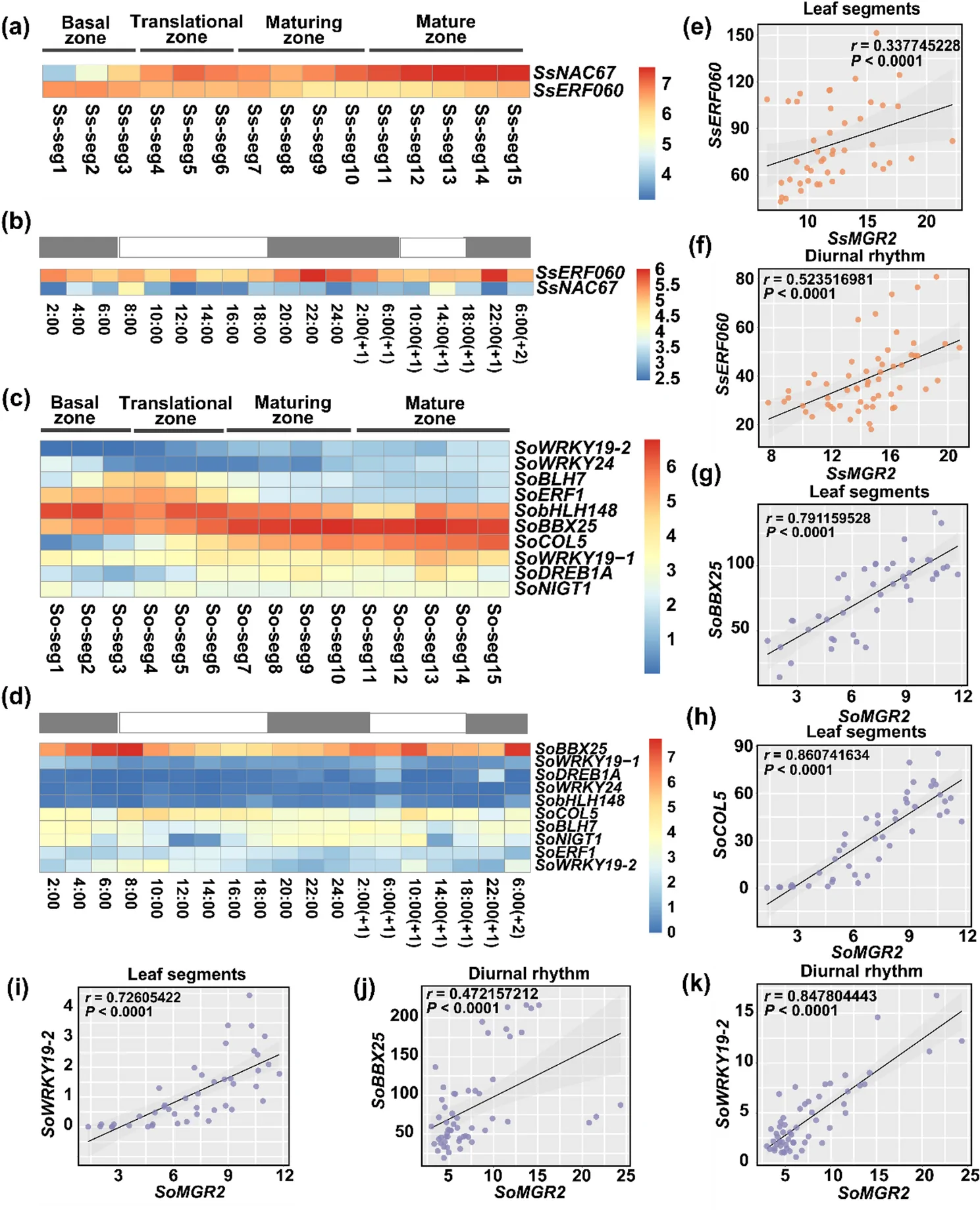
The correlations of the gradient development leaf segments and diurnal rhythm expression between TFs and *MGR2. * (**a**) Heatmap of the expression profile of TFs based on TPM in the gradient development leaf segments in *Ss*; (**b**) Heatmap of the expression profile of TFs based on TPM in diurnal rhythm in *Ss*; (**c**) Heatmap of the expression profile of TFs in the gradient development leaf segments in *So*; (**d**) Heatmap of the expression profile of TFs in the gradient development leaf segments and diurnal rhythm in *So*; (**e**-**k**) The correlations between TFs and *MGR2* expression in diurnal rhythm in *Ss* and *So*

Correlation analysis of TF and *MGR2* expression revealed distinct regulatory relationships between the two species. In *S. spontaneum*, the expression of *SsERF060* showed a strong positive correlation with that of *SsMGR2* across leaf developmental gradients and diurnal cycles, suggesting that SsERF060 is a key positive regulator of *SsMGR2*. In contrast, in *S. officinarum*, *SoMGR2* expression was strongly positively correlated with those of *SoBBX25*, *SoCOL5*, and *SoWRKY19*-2, while other transcription factor genes exhibited negative or weak correlations (Fig. 6; Supplementary Fig. 5).

**Fig. 7:**
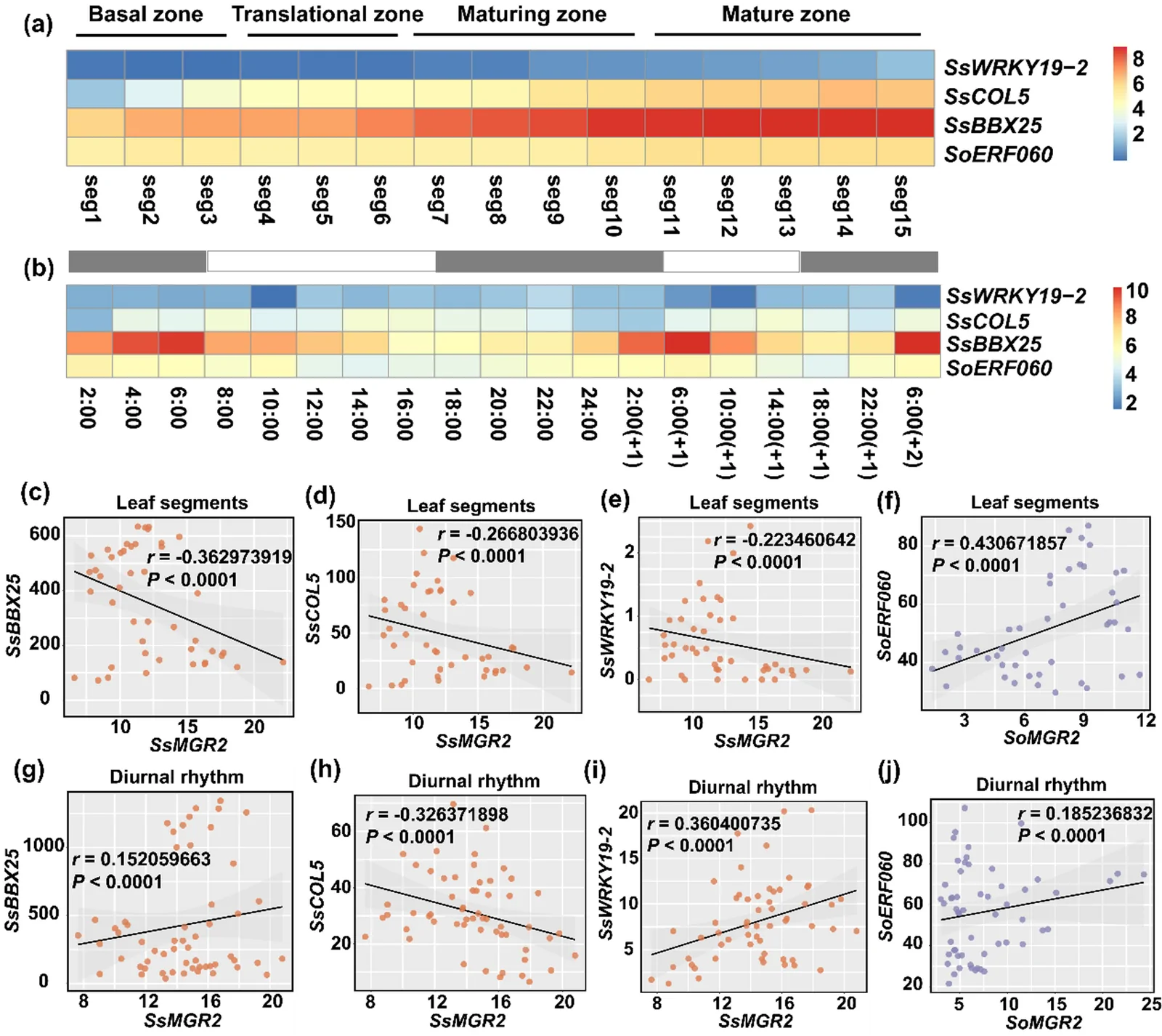
Expression analysis of homologous TFs across two species. (**a**) Heatmap of the expression profile of TFs based on TPM in the gradient development leaves; (**b**) Heatmap of the expression profile of TFs based on TPM in diurnal rhythm; (**c**-**f**) The correlations between TFs and *MGR2* expression in the gradient development leaf segments in *Ss* and *So*; (**g**-**j**) The correlations between TFs and *MGR2* expression in diurnal rhythm in *Ss* and *So*

Comparative analysis of homologous transcription factors further demonstrated that regulatory relationships between *MGR2* and upstream TFs are not conserved between two species (Fig. 7). Collectively, these results indicate that although *MGR2* retains a conserved Mg²⁺ transport function, its transcriptional regulation has diverged substantially between *S. spontaneum* and *S. officinarum*. This divergence in regulatory architecture likely contributes to species-specific Mg²⁺ responsiveness and differential adaptation to magnesium availability.

## Discussion

Mg²⁺ is indispensable for plant physiology, contributing to chloroplast biogenesis, osmotic regulation, and the activation of numerous enzymes [4, 32, 45]. Plant MGR proteins, first identified in *Arabidopsis thaliana* as homologs of the CorC/CNNM family, mediate Mg²⁺ flux across cellular membranes [25]. Although *MGR* genes have been characterized in several model plants, their biological roles and regulatory mechanisms in sugarcane remain largely unknown.

In this study, we systematically analyzed the *MGR* gene family in *S. spontaneum* and *S. officinarum*. Phylogenetic relationships revealed that sugarcane *MGR* genes are highly conserved with those in major monocot species such as *S. bicolor*, *Z. mays*, *S. viridis*, *B. distachyon*, and *O. sativa*. Notably, SsMGR1/SoMGR1 grouped with AtMGR1-AtMGR3 and OsMGR1, which localize to the vacuolar membrane and regulate Mg²⁺ sequestration [28], while OsMGR3 also facilitates Mg²⁺ extrusion from aleurone cells [27]. SsMGR2/SoMGR2 and SsMGR3/SoMGR3 cluster with AtMGR4–AtMGR7, known plasma-membrane transporters involved in root-to-shoot Mg²⁺ translocation [25]. SsMGR4/SoMGR4 grouped with AtMGR8-AtMGR9, implicated in chloroplast Mg²⁺ uptake [16]. These evolutionary patterns strongly suggest functional divergence among sugarcane MGRs, warranting further experimental validation.

Mg²⁺, as the central atom of chlorophyll and a cofactor for photosynthetic enzymes, profoundly influences photosynthetic differentiation along the leaf developmental gradient in sugarcane [46–49]. Moreover, Mg²⁺ homeostasis contributes to circadian rhythm stability, and the rhythmic expression of multiple Mg²⁺ transporters—such as OsMGT3 [50], OsMRS2-6 [51], and ZmMGT12 [52]- suggests the circadian clock may regulate Mg²⁺ transport [53]. By profiling *MGR* gene expression along sequential leaf segments and across diurnal cycles, we uncovered notable differences between the two species, particularly in *MGR2*. This species-specific expression pattern indicates possible divergence in regulatory pathways controlling Mg²⁺ homeostasis.

To functionally validate *MGR2*, we employed the *Salmonella typhimurium* MM281 mutant, which lacks the CorA, MgtA, and MgtB Mg²⁺ transport systems and fails to grow under Mg²⁺ limitation [21]. Both SsMGR2 and SoMGR2 restored MM281 growth under low-Mg²⁺ conditions, confirming their Mg²⁺ transport activity. This is consistent with previously characterized plant Mg²⁺ transporters, such as AtMGT1, AtMGT5, AtMGT10 [19], and AtMGR homologs [25]. Further, *SsMGR2* overexpression enhanced seedling fresh weight in rice under normal Mg²⁺ supply and improved chlorophyll content and biomass under low Mg²⁺ concentrations. Under excessive Mg²⁺ (10 mM), both wild-type and transgenic plants exhibited growth inhibition. However, *SsMGR2*-overexpressing plants maintained a relatively higher biomass. This suggests enhanced tolerance to Mg²⁺ toxicity. This phenotype resembles Arabidopsis *mgr4* and *mgr6* mutants, which modulate root-to-shoot Mg²⁺ loading to prevent excessive accumulation [25]. Thus, *SsMGR2* may similarly regulate Mg²⁺ distribution, a hypothesis requiring further investigation.

Promoter analyses revealed substantial differences in cis-regulatory landscapes between *SsMGR2* and *SoMGR2* (Supplementary Fig. 3). Since TF-cis-element interactions orchestrate precise spatial-temporal gene expression [54], we examined potential upstream regulators of *MGR2*. BBX25, COL5, and WRKY19-2 displayed species-specific regulatory patterns (Fig. 6), which may explain the divergent expression of *MGR2* between *S. spontaneum* and *S. officinarum*. Although these TFs are known to regulate stress responses, growth, photoperiod, and light morphogenesis [55, 56], their involvement in Mg²⁺ homeostasis is largely unexplored. Interestingly, the WRKY family participates in metal transport; for example, WRKY46 regulates VITL1 to modulate Fe²⁺ translocation under iron deficiency [57]. This suggests that WRKY and other TFs may similarly influence Mg²⁺ transport, highlighting promising candidates for further functional validation.

In summary, we performed the first comprehensive analysis of the *MGR* gene family in the two founding *Saccharum* species. We revealed species-specific expression divergence of *MGR2*, demonstrated its Mg²⁺ transport capacity, and identified TFs that may differentially regulate *MGR2* in *S. spontaneum* and *S. officinarum*. These findings provide new insights into Mg²⁺ homeostasis in sugarcane and establish a foundation for understanding the molecular mechanisms of interspecific variation in Mg²⁺ transport and regulation. Future studies should further investigate the regulatory mechanisms underlying *SsMGR2* and *SoMGR2* expression and clarify how *cis*-regulatory and TF networks contribute to species-specific Mg²⁺ responses. In addition, although this study validated the Mg²⁺ transport activity of MGR2 and identified species-specific transcriptional differences, further analyses including Dual-LUC assays, EMSA experiments, and photosynthetic physiological characterization will be necessary to fully elucidate the upstream regulatory mechanisms and downstream physiological consequences of MGR2-mediated Mg²⁺ homeostasis.

## Supplementary Information


Supplementary Material 1.



Supplementary Material 2.



Supplementary Material 3.



Supplementary Material 4.



Supplementary Material 5: Fig S1. *S. spontaneum (Ss)* and *S. officinarum (So)* MGR genes expression patterns. Fig S2. Identification and Phenotype of Rice with overexpression of *SsMGR2*. Fig S3. Analysis of *SoMGR2* and *SoMGR2* promoter-specific cis-acting elements. Fig S4. Screening of the minimum inhibitory concentration of AbA for the *SsMGR2* and *SoMGR2* promoter fragments. Fig S5. The correlations of gradient developmental leaf segments expression between TFs and *MGR2*.


## Data Availability

The data underlying this article were available in the National Center for Biotechnology Information (NCBI) short read archive (SRA) repository under the accession number PRJNA685968, PRJNA1200917 and PRJNA1436806.
