# Supplementary material for: Distinct Mg²⁺ responses and species-specific transcriptional regulation of the magnesium transporter MGR2 in Saccharum officinarum and Saccharum spontaneum
Source: BMC Plant Biol. 2026 Jun 18;26:1476. doi: 10.1186/s12870-026-09288-x (PMC13523378; doi:10.1186/s12870-026-09288-x)
Supplement: Supplementary file 1 [file 12870_2026_9288_MOESM5_ESM.docx]

**Supplementary Fig 1. *Ss* and *So* *MGR* genes expression patterns**

**a.** *MGR* genes expression patterns based on TPM in different development period of different organizations; **b.** *SsMGR*2 and *SoMGR2* expression patterns in different development period of different organizations; **c.** *MGR* genes expression patterns in gradient developmental leaf segments; **d.** *SsMGR*2 and *SoMGR2* expression patterns in gradient developmental leaf segments; **e.** *MGR* genes expression patterns based on TPM in diurnal rhythm; **f.** *SsMGR*2 and *SoMGR2* expression patterns based on TPM in diurnal rhythm.

**Supplementary Fig 2. Identification and Phenotype of Rice with overexpression of *SsMGR2***

a. The expression level of the *SsMGR2* gene in the overexpression lines of rice was identified by RT-qPCR; b. Phenotype of rice with overexpression of *SsMGR2*, with a scale of 20 cm.

**Supplementary Fig 3. Analysis of *SoMGR2* and *SoMGR2* promoter-specific cis-acting elements**

**a.** Analysis of the cis-elements in the 2 kb promoter region of the *SsMGR2*; **b.** Analysis of the cis-elements in the 2 kb promoter region of the *SoMGR2*.

**Supplementary Fig 4. Screening of the minimum inhibitory concentration of AbA for the *SsMGR2* and *SoMGR2* promoter fragments**

**Supplementary Fig 5． The correlations of gradient developmental leaf segments expression between TFs and *MGR2***

**a.** The correlations between TFs expression in gradient developmental leaf segments in *Ss*; **b-h.** The correlations between TFs expression in gradient developmental leaf segments in *So*; **i.** The correlations between TFs expression in diurnal rhythm in *Ss*; **j-q.** The correlations between TFs expression in diurnal rhythm in *So*. The red triangle indicates a strong positive correlation between the expression of TF and *MGR2*. The blue triangle indicates a strong negative correlation between the expression of this TF and *MGR2*.
